# Implementation in the “real world” of an evidence-based social and emotional learning program for teachers: effects on children social, emotional, behavioral and problem solving skills

**DOI:** 10.3389/fpsyg.2023.1198074

**Published:** 2024-02-21

**Authors:** Maria Filomena Gaspar, Maria Seabra-Santos, Joana Relvão, Mariana Pimentel, Tatiana Homem, Andreia Fernandes Azevedo, Mariana Moura-Ramos

**Affiliations:** ^1^Faculty of Psychology and Educational Sciences, University of Coimbra, Coimbra, Portugal; ^2^Centre for Social Studies, University of Coimbra, Coimbra, Portugal; ^3^Center for Research in Neuropsychology and Cognitive and Behavioral Intervention, University of Coimbra (CINEICC), Coimbra, Portugal; ^4^Clinical Psychology Unit, Centro Hospitalar e Universitário de Coimbra, Coimbra, Portugal

**Keywords:** social and emotional learning, school-based SEL intervention, fidelity, The Incredible Years^®^ Teacher Classroom Management, Academias Gulbenkian do Conhecimento, implementation science

## Abstract

**Introduction:**

The delivery of social and emotional learning (SEL) programs that are developmentally school-based and evidence-based has the potential to benefit many children, and as such, greater efforts are needed to disseminate these programs more widely within the community. The Incredible Years® Teacher Classroom Management (IY-TCM) has shown promising results when applied by teachers in preschool centers and primary schools, as seen in several randomized control trials conducted worldwide, including in Portugal.

**Methods:**

The current study presents a model of the implementation of the program within the framework of a nationwide initiative undertaken in Portugal: the Academias Gulbenkian do Conhecimento. Additionally, results of the program’s impact on children were explored using ANOVA, which compared pre- to post- treatment outcomes. To assess which factors affected the efficacy of the intervention, moderation analyses were conducted using the MEMORE macro. Ninety teachers and 535 children (2 to 10 years old) were assessed.

**Results:**

Results revealed that children showed significant increases in social and emotional skills (e.g., social adjustment, empathy) and significant reductions in problem behavior when assessed by their teachers, and in social-cognitive problem solving strategies as evaluated by a set of problem-solving tasks. Moderation analyses showed that, in general, interaction effects were not found, meaning that the intervention was effective for almost all conditions. Nevertheless, significant moderation effects were found for factors pertaining to the child and the mother with respect to pro-social and emotional skills (children who benefited most from the intervention exhibited more behavioral difficulties at the baseline according to the teachers’ perceptions and had mothers without a university degree; children attending primary school took less benefit from the intervention than those attending pre-school).

**Discussion:**

The findings contribute both to the reinforcement of the effectiveness of the IY-TCM program as a universal intervention in “real world” schools and to the development of some guidelines for the promotion of effective scaling up and sustainability of program effects.

## Introduction

Schools constitute a “universal access point” (Sanders et al., 2022, p. 949) from which interventions can be implemented to promote both the cognitive, emotional and social development of children and youth and their mental health. These interventions involve not only the children and youth in question, but also their families and the local communities (Clarke, 2019). As stated in the report entitled “Reimagining our future together” produced by the UNESCO International Commission on the Futures of Education (UNESCO, 2021, p. 4), schools have to be “protected educational sites because of the inclusion, equity and individual and collective well-being they support—and also reimagined to better promote the transformation of the world towards more just, equitable and sustainable futures.” In assuming this role, they become central in the efforts to achieve some of the 17 United Nations Sustainable Development Goals (SDGs) (UNESCO, 2021; Sanders et al., 2022).

School-based interventions to promote social and emotional development, encompassed in the macro concept of “Social and emotional learning” (SEL), can be classified into 3 types, according to Clarke (2019): (1) whole-school intervention targeting the school as a whole and integrating a coordination between curriculum, school and family and community partnerships; (2) universal classroom skills-based intervention, for all students in a classroom; (3) targeted intervention, concentrating on students who present different types of risk factors that may compromise their mental health and well-being. The focus of this paper is the implementation of an evidence-based intervention, the Incredible Years^®^ Teacher Classroom Management program (IY-TCM; Webster-Stratton, 2011a, 2012), at the classroom level for all children, even though it features contents that may address the specific needs of certain students (e.g., individual behavioral plans enabling the teacher to work with children who present more socioemotional difficulties in the classroom, involving their families and other school-based professionals). In this way, the program integrates the recommendation of “proportionate universalism” (Sanders et al., 2022, p. 945; Barry, 2019a, p. 38), as far as it is universal and inclusive, yet “calibrated proportionally” to the level of need or disadvantage (World Health Organization and Calouste Gulbenkian Foundation, 2014, p. 8).

Studying the implementation of evidence-based practices (EBP) in real-world schools is essential to informing successful implementation, and thus improving students’ outcomes as intended and decreasing not just the “research-to-practice gap” in education (the EBP be adopted) but also the “implementation gap” (the EBP be implemented in schools routinely as planned) (Hagermoser Sanetti and Collier-Meek, 2019).

As Shonkoff (2017) stated in a commentary about the outcomes of early childhood interventions, not only have few programs been scaled effectively, but their effects also appear small to moderate with respect to important dimensions of child development. He thus argues that we need to redefine the criteria we use to classify a program as “evidence-based,” removing the focus only from the analysis of statistically significant differences between a control group and an experimental group in randomized studies, and placing it more on causal models focused on mediating and moderating variables—that is, the “on-the-ground experience”—so that they can more effectively answer the questions focusing on which contexts, whether, for whom and to what extent the interventions achieve the intended effects (Shonkoff, 2017).

According to Proctor et al. (2011), it is essential to distinguish “treatment effectiveness” from “implementation effectiveness” in order to transport evidence-based practices or innovations to the community and services and to assess when failure occurs, whether it is due to the intervention’s ineffectiveness in that context (intervention failure) or its incorrect implementation (implementation failure). On the assumption that “a critical yet unresolved issue in the field of implementation science is how to conceptualize and evaluate success” (Proctor et al., 2011, p. 65), they proposed a model to assess implementation success centered on what they called “implementation outcomes,” which precede and are different from service system outcomes (e.g., effectiveness) and customer outcomes (e.g., satisfaction). Implementation outcomes encompass the effects of actions that have specific objectives and are undertaken intentionally in the implementation of new services, interventions, or practices. The authors developed an “implementation outcomes taxonomy” including eight different outcomes:

(1) Acceptability (satisfaction with aspects of the innovation); (2) adoption (initial decision or utilization or intention to try); (3) appropriateness (usefulness, perceived fit); (4) feasibility (practicability, suitability for use); (5) fidelity (i.e., delivered as intended by program developers, which includes: adherence to the program protocol, dosage, and quality of program delivery); (6) implementation cost (cost-benefit, cost-effectiveness); (7) penetration (integration at the level of the organization or setting); (8) sustainability (sustained use, maintenance, integration within the organization’s culture).

The overarching aim of this paper is to document a model of implementation of an evidence-based SEL program, the IY-TCM, in real-word, school-based settings (preschools and primary schools in Portugal) under a broader national innovation initiative developed with the purpose of promoting the social and emotional competences of children and young people aged 25 and under: The Academias Gulbenkian do Conhecimento initiative of the Fundação Calouste Gulbenkian. Another objective is to assess implementation success through the effectiveness of the IY-TCM on improving children’s social, emotional, behavioral and problem solving skills and considering different types of moderators: level of teachers’ IY-TCM training (at the local community level by no experienced group-leaders from local entities; at the university level by experienced group-leaders); professional background of the participants involved in the program’s implementation with children (teachers versus other school-based professionals); educational system level of the classrooms where the intervention was implemented (preschool versus primary school); mother’s level of education (primary or lower secondary; upper secondary; university degree); teachers’ perceptions about the children’s behavior (easy/average or difficult).

### Study background

#### The Academias Gulbenkian do Conhecimento

The Fundação Calouste Gulbenkian (FCG) is a Portuguese private philanthropic institution whose main purpose is improving the quality of life through initiatives that support the arts, charitable endeavors, science and education.^1^ In May 2018 the FCG launched an initiative—The Academias Gulbenkian do Conhecimento. The academies are institutional consortiums, involving non-profit public or private or social sector organizations, including, but not limited to, youth, cultural, and sports associations, NGOs, private social solidarity institutions, parents’ associations, municipalities, schools, universities, and hospitals responsible for the implementation of projects (“methodologies”) that would promote the social and emotional competences of children and young adults up to 25 years of age. Calls for proposals were opened in three consecutive years (2018, 2019 and 2020) with 100 projects, in different fields (culture, education, sports, health, solidarity or technology) selected and funded in every region of Continental Portugal and the autonomous regions of Madeira and the Azores.

Seven social and emotional competences were considered to be fundamental for children and young adults up to 25 to deal with sudden life changes, and were thus selected as the focus for the interventions^2^:

- Adaptability: adjusting to change by flexibly adapting their attitudes and behaviors;

- Self-regulation: being decisive, strategic and persistent in goals, evaluating progress and modifying behaviors as a result of that evaluation;

- Creativity: having a vision and generating new ways of thinking and doing, exploring and learning from error;

- Problem solving: realistically assessing problems, looking for alternatives, deciding and implementing solutions using creativity and logical thinking, keeping in mind the consequences for oneself and others;

- Critical thinking: valuing situations from multiple perspectives, breaking down problems into their components, and systematizing the path to resolution through new methods and processes, looking for causes or thinking through the consequences of the various possible courses of action;

- Resilience: handling adversity well and not giving up easily;

- Communication: initiating and maintaining social contacts, expressing opinions, needs or feelings appropriately.

Each academy applying for funding had to demonstrate how its project would contribute to the development of some of these seven competencies.

The academies could choose to apply to the implementation of one of two types of interventions (“methodologies”): (1) “reference methodology” selected *a priori* by the FCG and which had already proven its effectiveness in Portugal (a total of nine different methodologies in the three calls)^3^; (2) “experimental methodology,” a new methodology whose effectiveness the academy wants to evaluate. The present paper is based on the work done within academies that used the Incredible Years^®^ Teacher Classroom Management (IY-TCM), which was one of the three reference methodologies proposed in the first call.

#### The Incredible Years^®^, Teacher Classroom Management Program

##### The program: content, processes, implementation

The Incredible Years^®^ Teacher Classroom Management (IY-TCM), one of the programs of Incredible Years^®^ (IY) series of programs for teachers, parents and children, was developed by Webster-Stratton to support teachers of children aged 3 to 8 years to effectively manage the disruptive behavior in their classrooms by promoting socio-emotional learning and a positive relationship with children and their parents (Reinke et al., 2012). It has thus been classified as a SEL program (Sandilos et al., 2020) grounded in both social learning and coercion theories (McClelland et al., 2017), but also in attachment theory (Tveit et al., 2020) because of the strong emphasis it places on the quality of the teacher’s relationship with the child. The program is organized around the following content components: strengthening of the teacher-student bond and home-school collaboration; classroom management skills, proactive teaching, effective discipline; academic persistence, social and emotional coaching with students; teaching social skills, anger management and problem-solving skills in class; individual behavior plans for children who exhibit some behavior difficulties; and building teacher support networks (Webster-Stratton, 2012).

The IY author developed a model of professional training and coaching that incorporates a guarantee of fidelity that increases the likelihood of implementation success. In fact, group leaders (or facilitators) who will deliver the program to teachers need to complete a 3 days training workshop, certified by the Incredible Years^®^, while participation in regular supervision with a coach or mentor in the program is also highly recommended by the author (Webster-Stratton and Bywater, 2015). Group-leaders training workshops can only be offered by “mentors” or “trainers” who themselves have followed a consistent training program that includes being certified as group-leader, having considerable experience delivering the program, and having completed training in coaching, supervision and workshop delivery skills (see https://incredibleyears.com/programs/implementation/ for more details). Mentors provide ongoing mentoring and supervision to group-leaders and work closely with the program author and participate regularly in international IY mentor meetings to improve their skills and guarantee they are familiar with and integrate in their trainings the latest improvements the author has introduced into the program content and processes.

The program is implemented by two trained group-leaders to groups of 14–16 preschool or primary school teachers, or other professionals working with children in educational environments, and is supported in a detailed Leader’s Manual (Webster-Stratton, 2011b) and books. The training model integrates a collaborative, self-reflective, and experiential learning process, in which teachers share ideas, role-play practices and discuss and problem-solve situations presented on DVD vignettes (Webster-Stratton, 2011a). In each training session teachers are invited to set personal goals from a self-monitoring checklist and to complete a self-reflection inventory. Between sessions group-leaders offer individual support to teachers, both online and in their classrooms, to help them solve/reflect on implementation issues and other problems and support them in implementing the strategies. Teachers are stimulated to share experiences and ideas with other teachers both between sessions and at the beginning of each session, with the goal of building teacher support networks and promote peer to peer learning (Webster-Stratton, 2011b).

The model for teacher training recommends 42 to 48 h of training in six one-day monthly workshops, implemented throughout the school year (Webster-Stratton, 2012). However other implementation models are used with efficacy. For example, Carlson et al. (2011) reported eight 4 h sessions over an 8–10 weeks period for a total of 32 h of training, and Gaspar et al. (2022) reported six 6 h workshops once a month or every 3 weeks, interspersed with 2 hours individual in loco peer coaching. According to Korest and Carlson (2022), dosage should be calculated not considering the number of sessions, because of the varied number of sessions offered, but rather by the number of hours, coding as “high dosage” if the training offered lasts at least 42 h.

##### The IY-TCM as an evidence-based program

In different countries, the IY-TCM as a stand-alone school-based intervention showed promising benefits for both children and teachers. Results from a very recent meta-analysis—one designed both to assess the current state of evidence in improving teachers’ and children’s outcomes and to identify potential intervention moderators of the effects of the IY-TCM as a stand-alone program (Korest and Carlson, 2022)—revealed the program had moderate positive effects on teachers (use of positive and negative IY-TCM classroom management strategies) with larger effect sizes in higher dosage studies (training hours offered greater than or equal to 42 h). Considering the effects on children, the results indicated small positive effects on children’s externalizing behavior and prosocial skills for teacher-rated reports, with larger effect sizes for higher risk children (behavioral problems above the clinical range defined by the study). The severity of child behavior (high risk and low risk), reporting methods (observation and teacher-rated), study design [randomized control trials (RCT) or quasi-experimental] and dosage (high = training hours offered greater than or equal to 42 h; low = less than 42 h) were the moderators analyzed, but because of the small sample only descriptive versus empirical analysis was possible. So the moderation results reported need to be read with caution. Sixteen studies (with a RCT or quasi-experimental design) from six countries (United States, United Kingdom, Ireland, Portugal, New Zealand, and Jamaica) were included.

In a previous mixed methods systematic review (Nye et al., 2019), the authors concluded that the program has the potential to provide a scalable public health solution to address both teachers’ needs related with classroom management problems and children’s social, emotional and behavioral needs, both in high-income countries (England, Ireland, Wales, United States) and in low-income countries (Jamaica). Results indicate a reduction in school violence related both with a reduction in teachers’ use of negative strategies, and with the improvement in the behavior of higher risk children in the classroom.

The IY-TCM is listed in online registries hosted by government and non-governmental organizations and designed to inform investment decisions by policy makers and commissioners (e.g., Blueprints for Violence Prevention Model and Promising Programs, administered by the Center for the Study and Prevention of Violence at the University of Colorado; https://www.blueprintsprograms.org/; The European Platform for Investing in Children (EPIC), an evidence-based online platform that provides information about policies that can help children and their families face the challenges in the current economic climate in Europe; https://ec.europa.eu/social/main.jsp?catId=1246&langId=en).

In Portugal the first study with the IY-TCM was a universal prevention quasi-experimental study conducted within the scope of a doctoral dissertation (Vale, 2012). Its main aim was to establish preliminary evidence on the program’s effectiveness in improving Portuguese children’s social skills and behavioral difficulties at school and teacher practices and behaviors. A secondary aim was to assess its acceptability by teachers. Changes happened in the expected direction and were sustained over time (12 months follow-up) regarding both children’s outcomes (including children with early signs of disruptive behavior), and teachers outcomes. High levels of teacher satisfaction with numerous aspects of the program were found. However, concerning the video clips, although teachers recognized their usefulness for stimulating discussion and modeling certain strategies, they thought that the videos did not adequately reflect the reality of young learners in Portuguese classrooms and therefore needed to be adapted (Vale, 2012). Seabra-Santos et al. (2018) conducted an RCT aiming to analyze the impact of the IY-TCM on social skills and behavior problems of economically disadvantaged preschoolers. After their teachers attended the IY-TCM training, children from the experimental group were rated with more social skills and fewer behavior problems. Moreover, higher improvements in social skills were found in children from economically disadvantaged families and with children at high risk because of their lower social skills. Within the same study, Gaspar et al. (2022) reported that teachers who participated in the IY-TCM showed an increased use of classroom management positive strategies and a reduced use of inappropriate ones. An impact on psychological variables was not found.

Considering that one of the key principles of practice to be followed in the implementation of innovations promoting mental health interventions is the selection of theoretical and evidence-based interventions (Barry, 2019b), the adoption of the IY-TCM by the FCG as a “reference methodology,” whose implementation in Portugal they supported and funded, seems justified.

## Method

### Implementation design

To more fully inform those applying for the Academias Gulbenkian do Conhecimento 2018 grant as to the specific components and goals of the IY-TCM, it was natural that the promoter should approach the team responsible for the implementation and research of the IY-TCM in Portugal, based at the Faculdade de Psicologia e de Ciências da Educação, Universidade de Coimbra (UC), to write a manual with the details of the intervention and implementation model (program contents, processes and goals; group-leader training; training to teachers and other school-based professionals who work with children; implementation support by the research team; outcomes and processes assessment model; program efficacy and effectiveness world-wide and in Portugal related with the expected results of the Academias Gulbenkian do Conhecimento in terms of improvement of social and emotional competencies of children) (The manual, in Portuguese, can be found in https://cdn.gulbenkian.pt/academias/wp-content/uploads/sites/43/2018/05/1.incredible_years.pdf).

The goal was to support applicants’ informed selection of the methodology, considering their own needs and resources. This is particularly relevant because in the 2018 call the applicants could choose among either four “reference methodologies” or the implementation of a methodology selected by themselves (“experimental methodologies”).

Seven applicants had their projects to implement the IY-TCM program approved. These academies had outlined projects with a variable duration, ranging from 12 to 36 months of implementation, which could be carried out either in preschools or in primary schools, and involved one of the following levels of training, or both: level (1) the teachers or other classroom-based professionals are trained by group-leaders from the academy who, in turn, have been trained by a program mentor from the university team; level (2) the teachers or other classroom-based professionals who will use the program with children in classrooms are trained directly by group-leaders from the university team. One of the academies chose to implement a project involving the two levels of training.

The implementation plan of the IY-TCM methodology followed 4 sequential steps:

Step 1. Formal agreement between the FCG and the UC concerning the tasks and duties of each one and the funding the former gives to the latter to do the training and provide the support needed for the successful implementation of the projects of the seven IY-TCM academies and also to conduct an evaluation of the implementation process and success.

Step 2. Face to face meeting between the promotor Agency (FCG—Academias Gulbenkian do Conhecimento), the coordinator Agency (UC), and the local Agencies coordinators (IY-TCM academies). The coordinator from the university presented the model of implementation of the IY-TCM methodology, the implementation support offered to academies and the assessment model of the implementation.

Step 3. Training of group leaders: only for the level 1 academies. Twenty-five professionals from the four level 1 academies (A1, A2, A3 and A4) participated in the 3-day leaders’ training workshop at the UC. The training was delivered by two group leaders with extensive experience with the IY programs, one of whom was a mentor in training of the IY-TCM. The training followed the same collaborative model that the trainees were supposed to use when running teachers’ groups.

During and after the certified training, the academies were closely supported by the university team: (1) to order the Portuguese version of the IY-TCM program materials (e.g., DVD, group leader manuals); (2) to establish a partnership with a local Center for Continuing Professional Development for Teachers, so that the teachers attending the IY program training might obtain professional credits for participating (given that in Portugal all the teachers, including preschool teachers, are encouraged to do certified continuing professional training in order to get professional credits to progress in their career); (3) to disseminate the IY-TCM program and the project in the local schools to recruit teachers that would volunteer to attend the program. Models of formal letters to the directors of school clusters, head teachers and teachers were made available.

Step 4. Program implementation to groups of teachers and other school-based professionals who work with classrooms: year 1.

#### Level 1 academies

The four academies disseminated the program in local community schools clusters, implementing it in schools to groups of teachers or other school-based professionals who worked with children in the classrooms. All teachers received professional credits for completing the program. All the four academies offered the 42 h of training in 7 monthly sessions of 6 h each, or in 14 sessions of 3 h each every 2 weeks. In the first year of implementation, all the workshops were administered in person; however, following the COVID-19 pandemic, two of the academies started to deliver online as well.

Before and during the first year of implementation, all the professionals trained in level 1 received the support from a member from the IY-TCM team based at the university. At least one supervision session took place face-to-face, which was attended either by all the group-leaders from the academy or from two academies in geographical proximity. Group-leaders were invited to take self and peer evaluations to the supervision session along with the evaluations of each session made by the teachers, at which point the collaborative problem-solving model recommended by the program author was followed. Online supervision sessions were also implemented with the same goal. At the end of the first year, after all the academies had finished the implementation of their first group, all the group-leaders were invited to participate in a focus group at the UC, in September 2019, to explore their views on the program’s strengths, its impact on teachers, any barriers they faced in the implementation, and suggestions for sustainability. One of the four academies finished the project, under the Academias Gulbenkian do Conhecimento, at the end of the school year 2018–2019 (June 2019) whereas the other three concluded in the 2020–2021 school year.

#### Level 2 academies

A group of 20 teachers and other school-based professionals from four academies (one is also a level 1 academy) participated in the teacher training led by two group-leaders from the university team. The training was implemented in seven full-day (6 h) workshops, occurring monthly during the school year. All the sessions took place on Saturdays at the university facilities. In order to encourage participation all the teachers were given professional credits, and lunch and coffee-breaks were offered. Between sessions, group-leaders offered individual support to teachers in their classrooms (twice) or online (four times) to support them in implementing the strategies and help them to solve or reflect upon other problems they faced in the implementation. Both group-leaders received close support in training from the UC team mentor in terms of preparing the sessions, solving problems and implementing the training according to the collaborative model. Self and peer evaluations along with the participant’s evaluations of each session were completed and used to support the supervision. At the end of implementation, all the participants were invited for a focus group held at the UC in October 2019, with different goals from those emphasized with level 1 academies: to explore the acceptability of the program and their views about which elements offered barriers to or facilitated implementation in schools. The level 2 academies only took place in the first year of the Academias Gulbenkian do Conhecimento (2018–2019).

#### Online implementation

Because of the COVID-19 pandemic, the level 1 academies did not have the chance to implement the IY-TCM program during the 2019–2020 school year. However, following the guidelines developed by the program author regarding online implementation of the IY-TCM, they supported the teachers with whom they worked to use the contents and processes of the program during their online contacts with children and parents. After the COVID-19 pandemic, two of the academies started to deliver the program online, with one delivering a group in a mixed format as they had begun in person but later, because of the pandemic-related restrictions, were forced to continue online.

To support all group-leaders with online delivery of the program (including the ones who had finished the contract with the FCG at the end of the first year) the mentor from the UC team ran a 2 h online webinar in January 2021 to share recommendations and strategies developed by the program developer (see https://incredibleyears.com/resources/gl/resources-for-group-leaders-working-remotely/ for more details about IY-TCM online implementation).

### Intervention assessment

#### Procedures

**Figure 1:**
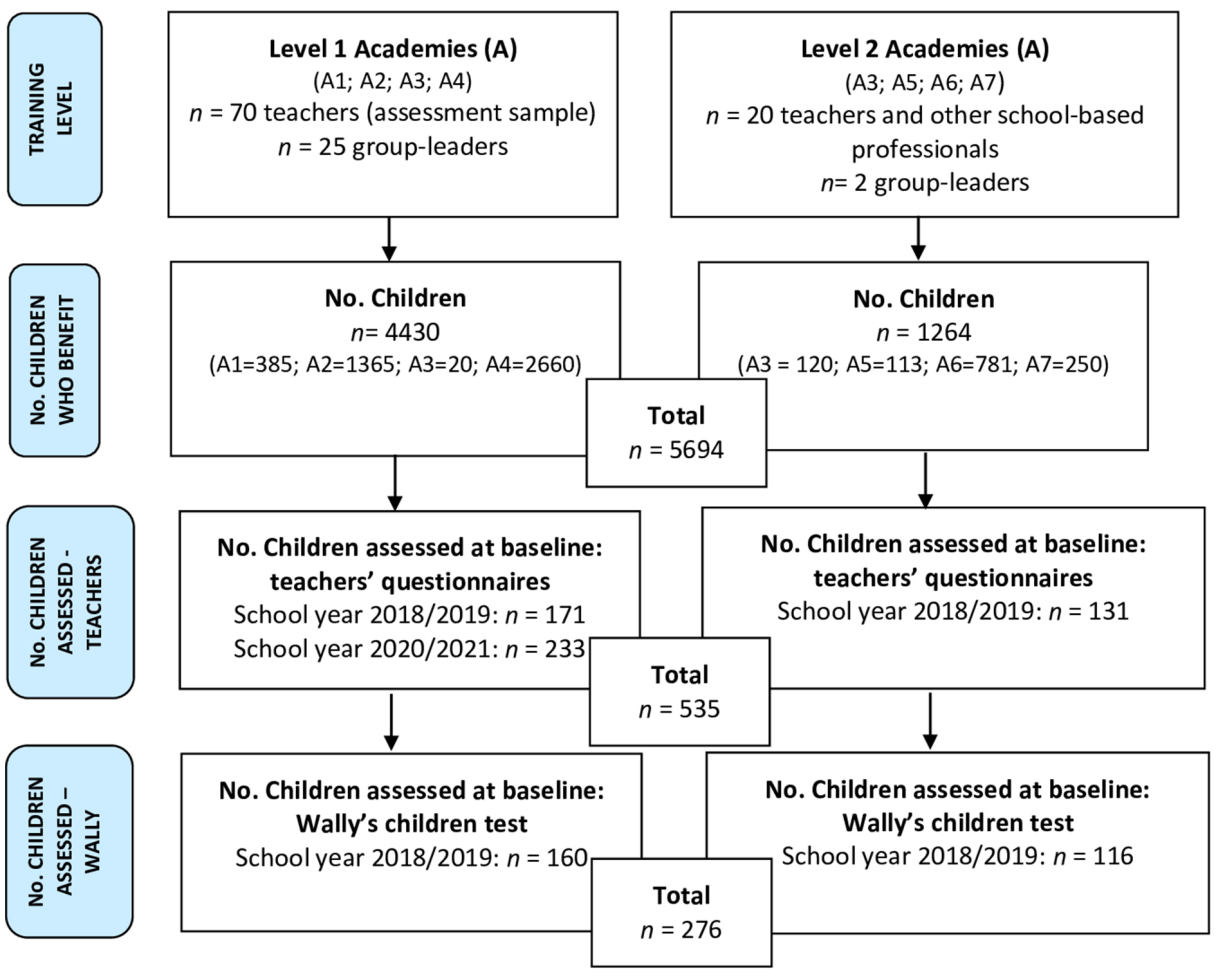
Flow chart of number of children who benefit from de intervention and who participate in the outcomes assessment according to the seven (A1 to A7) academies training level.

A total of 5,694 children were offered the IY-TCM program (cf. Figure 1, step 2). However, to examine the effectiveness of the program, two teachers were randomly selected from each of the groups in level 1, and all the teachers in level 2 participated (cf. Figure 1, step 1). Regarding the selection of the children for inclusion in the assessment, the method used was inspired in the procedures used by Leckey et al. (2016): each previously recruited teacher selected a total of six children from their classroom based on their evaluation of difficult behaviour. Two children considered to be “easy,” two considered to be “average” and two considered to be “difficult.” Therefore, although a total of 5,694 children benefited from the program, only a subsample of 9.4% were used for the purpose of assessing the effectiveness of the program presented here (cf. Figure 1, step 2 to step 3).

A written consent was signed by all participant teachers/professionals and parents. On a day previously agreed upon with the teachers/professionals involved in the assessment, two psychologists from the UC team with vast experience in the assessment of children went to schools to individually evaluate the six previously selected children (cf. Figure 1, step 4) and to ask teachers to answer the questionnaires concerning each one. Baseline assessment occurred at the beginning of school year immediately before the intervention started. Post intervention assessment was conducted in the end of the intervention, approximately 7 months after baseline.

#### Participants

##### Academies

The seven academies participated in the effectiveness assessment (cf. Figure 1).

Four are level 1 academies. A1 is a non-profit community agency with extensive experience in community work, including work with schools in the Lisbon area. A2 is a Department of Child and Adolescent Psychiatry, from a major hospital in the north of the country, strongly committed to mental-health prevention and with large experience in offering IY-Basic parent groups and with partnerships with teams from public health, local schools and the municipality. The group leaders were from different disciplines: psychology, health, social education, primary school and preschool education. A3 is a non-profit private preschool center in the south of the country that wants to bring the IY philosophy to all school staff, professionals and non-professionals. The director and another preschool teacher attended the group-leaders training (level 1) and four other preschool teachers participated in the level 2 training. A4 is a health department of a polytechnic university in the center area of the country. The group-leaders came from different disciplines: health, social education, psychology, and preschool education.

Three others are level 2 academies. A5 is a cluster of schools in the center of Portugal, which had six preschool and primary school teachers participating in the IY-TCM workshop. A6 is a local government service in the Lisbon area, which implemented the IY-TCM in preschool classrooms. As for their professional background, they were psychologists, educational specialists and one was a teacher. A7 is a non-profit organization in the Lisbon area and as A6 implemented the IY-TCM with children in preschool classrooms, their professionals were not teachers but had professional training in social, cultural and educational disciplines.

##### Teachers and other school-based professionals

Ninety professionals from 7 academies (cf. Figure 1, step 1) participated in the evaluation.

Table 1 presents some of their characteristics. Most of them were teachers (78%). Concerning the non-teaching professionals, seven were school or clinical psychologists and the others were from areas of education. All the professionals, including preschool and primary teachers, had at least a bachelor’s degree. They had worked as teachers for an average of 23.36 years (SD = 8.86).

**Table 1:** Children’s and educational professionals’ characteristics at baseline.

Children			Professionals		
*N* = 535			*N* = 90		
Age (years)	Min. = 2 Max. = 10	*M* = 5.66 *SD* = 1.90	Professionals’ education (*n*, %)		
				Teachers	78 (87%)
Level of schooling (*n*, %)	Preschool	321 (60)		Non-teachers	12 (13%)
	Primary school (1st to 4th year)	214 (40)	Teachers’ professional experience (years) (*n* = 78)	Min. = 4 Max. = 40	*M* = 23.36 SD = 8.86
Behavior (*n* = 529) (*n*, %)					
	Easy/average	355 (67.1)			
	Difficult	174 (32.9)			
Mother’s level of education (*n* = 408) (*n*, %)			Classrooms		
			*N* = 90		
	Basic (<=9 years)	107 (26.2)	Number of children in the classroom	Min. = 10 Max. = 26	*M* = 19.86 SD = 3.87
	Secondary (12 years)	124 (30.4)	Number of boys in the classroom	Min. = 5 Max. = 16	*M* = 10.29 SD = 2.59
	University degree	177 (43.4)	Number of girls in the classroom	Min. = 2 Max. = 16	*M* = 9.41 SD = 3.18
Father’s level of education (*n* = 375) (*n*, %)					
	Basic (<=9 years)	136 (36.3)			
	Secondary (12 years)	138 (36.8)			
	University degree	101 (26.9)			

##### Children

In each classroom, six children were selected to participate in the effectiveness study: teachers identified two children they considered to be “easy,” two “average” and two “difficult.” In this way 535 children aged 2 to 10 years (*M* = 5.66 years; SD = 1.90) participated in the intervention assessment. Table 1 presents the main sociodemographic characteristics of the sample. More children (60%) are in preschool classrooms compared to the ones in primary school (40%), with this last level corresponding to the first 4 years of compulsory education that in Portugal starts when children are 6 years old. Thirty-three percent were considered to be “difficult” by their teachers. Forty-three percent had mothers with a university degree and 26% had mothers with 9 or less years of education (basic education). The father’s education level was found to be lower than the mother’s.

### Measures

#### Teacher and other school-based professionals and classroom characteristics

A questionnaire was developed to collect data on the IY-TCM program participants (e.g., professional education, years of experience as teachers), as well as on the classroom characteristics (e.g., number of children, number of boys and girls). It also included some questions aimed at characterizing the six children in each classroom selected for the effectiveness study (e.g., age, mother’s and father’s level of education).

#### Children outcomes

##### Behavior problems

The Strengths and Difficulties Questionnaire (SDQ; Goodman, 1997; Portuguese version by Fleitlich et al., 2005) was used to evaluate children’s behavior problems. The SDQ is a 25-item inventory with different versions depending on the child’s age range (2 to 4 years-old and 4 to 17 years-old), and on whether the respondent is a parent, teacher or oneself (the latter only for children from 11 to 17). The questionnaire consists of five subscales including five items each: hyperactivity/inattention, emotional problems, conduct problems, peer problems, and prosocial behaviors. Each item is answered on a three point scale: “not true,” “somewhat true,” or “certainly true,” with a minimum score of 0, and a maximum of 10 for each subscale, from which different risk levels are defined. Scores on the first four subscales can be aggregated into a composite of total difficulties (with a minimum score of 0, and a maximum of 40), which is used in this study as an outcome measure. In the present study, the version intended for 4–17 years-old was completed by teachers, who provided answers reporting on the child’s behavior over the previous 6 months, as per the instructions. The internal consistency for the composite of total difficulties was 0.80 at baseline and 0.81 at post-intervention.

##### Social and emotional skills

Two questionnaires, answered by the children’s teachers, were used to evaluate the social skills of children, according to their school level, both authored by Merrell: The Social Skills Scale of the Preschool and Kindergarten Behavior Scales—Second Edition (PKBS-2; Merrell, 2002a; Major, 2011; Major and Seabra-Santos, 2014), and the Social Competence Scale of the School Social Behavior Scales—Second Edition (SSBS-2; Merrell, 2002b; Raimundo et al., 2012). For the present study, in order to achieve a common measure for both preschoolers and school aged children, the two scales were compared and the common items were retained for analysis: 6 items deal with Self-Management/Compliance (e.g., “Follows school and classroom rules”) and 4 items are related to Peer Relations/Empathy (e.g., “Offers help to other children when they need it”). Good internal consistency levels were obtained for both set of items: 0.91 and 0.87 for Self-Management/Compliance, and 0.87 and 0.88 for Peer Relations/Empathy, at baseline and at post-intervention, respectively.

##### Problem solving

The Wally Problem Solving test (Webster-Stratton, 1990) was administered to evaluate the children’s capacity to find solutions to challenging social situations. The original test presented 12 colored pictures showing social problem scenes that can typically arise in interactions with preschool or early elementary school peers or teachers, or at home with parents. The test version used in this study is a shorter form with six vignettes (Webster-Stratton et al., 2001), including two social challenges, two scenarios with a desired object, and two scenarios of potential punishment. The test was administered in a one-on-one interview format, during which children were shown each of the six images, with the main character matched to their gender and the situation described verbally. Children were then asked what they would do if they faced the social problem depicted and were encouraged to give additional solutions, limited to a total of six responses or until they stopped adding different content. The responses were coded according to the following three indexes, calculated across the six vignettes: (i) proportion of positive solutions, as an indicator of prosocial and self-regulated ways of solving problems; (ii) proportion of aggressive solutions, representing difficulties in the social relationships and self-regulation; and (iii) persistence of positive solutions, indicating the child’s capacity to persist in prosocial and positive solutions, before an aggressive solution is given as a response to the problem.

### Data analysis

Statistical analyses were performed using SPSS 27.0. Descriptive analyses were conducted to determine the demographic characteristics of the sample. Missing data was low level (<10%) and at random, so missing values were replaced by the mean of the subscale.

The effects of the intervention were analyzed using *t*-test statistics for paired samples comparing score at baseline and scores after the intervention. Considering that multiple comparisons were performed, we used the Bonferroni correction for multiple comparison. The level of significance considered was 0.008 (0.05/6). Cohen’s *d* for estimating the effect sizes was calculated using the Lenhard and Lenhard (2016) calculator. Cohen’s *d* effect sizes were interpreted considering a value of 0.2 for a small effect size, a value of 0.5 for a medium effect size and a value of 0.8 for a large effect size. *A priori* sample size calculations (Faul et al., 2007) revealed that for a power of 0.90, with significance level of 0.05, testing for differences between two means using *t*-tests, a minimum of 216 participants in the total sample was required for detecting small effects (*d* = 0.02).

Moderation analyses were conducted using the MEMORE (Montoya, 2019) macro for mediation and moderation analysis (model 2), which is a tool available for SPSS to estimate and probe interactions when the focal predictor is a within-participant factor. Examined moderators included variables related to the child, the level of teachers’ training in the IY-TCM, and the professional background of the teachers and other school-based professionals who implemented the IY-TCM in the classrooms. Regarding the moderation effects, GPower was also used for calculating sample sizes: for a power of 0.90, with significance level of 0.05, testing for linear multiple regression (fixed model, *r*^2^ increase), a minimum of 353 participants in the total sample was required for detecting small effects (*f*^2^ = 0.03).

## Results

### Intervention effects

Table 2 presents the means, standard deviations (SD) and the significance tests of the comparison between the baseline and the post intervention scores for all the study variables.

**Table 2:** Descriptives and pre to post intervention comparison of the outcome variables.

Outcomes	*N*	Baseline (mean ± SD)	Post intervention (mean ± SD)	*t*-test	*p*	Cohen *d*
*Children’s social and emotional skills*						
Self-Management/compliance	505	20.35 ± 3.69	21.45 ± 3.08	−10.81	<0.001	0.32
Peer-relations/empathy	508	13.49 ± 2.58	14.30 ± 2.16	−10.84	<0.001	0.33
*Behavior problems*						
SDQ total difficulties score	517	11.62 ± 6.47	10.45 ± 6.30	7.124	<0.001	0.18
*Social problem-solving strategies*						
Proportion of positive solutions	276	85.08 ± 17.88	88.81 ± 13.32	−3.76	<0.001	0.25
Proportion of aggressive solutions	276	3.59 ± 7.40	2.25 ± 5.67	−2.91	0.004	0.20
Persistence of positive solutions	276	81.62 ± 19.89	85.17 ± 14.42	−2.95	0.003	0.19

As presented in the table, significant changes were observed in all variables that were assessed. Children assessed before and after the intervention significantly increased their social and emotional skills, namely self-management/compliance and peer-relations/ empathy, and the effect sizes of these changes were small. Similarly, regarding social problem-solving strategies, there were significant increases from the baseline to the post intervention, also of small effect sizes. Finally, results also showed that children significantly decreased their scores in terms of behavior problems, although in the case the effect size was the smaller found (Cohen *d* = 0.18).

### Moderation effects of the intervention

Moderation effects were examined for all the outcome variables: children’s social and emotional skills (self-management/compliance; peer-relations/empathy), behavior problems (SDQ total difficulties score) and social problem-solving strategies (proportion of positive solutions, persistence of positive solutions, and proportion of aggressive solutions). Moderators that were tested were related to the:

(1) children’s characteristics (children’s behavior assessed by their teacher: 0 = easy/average, 1 = difficult; children’s level of schooling: 0 = preschool, 1 = primary school); (2) mother’s education (mothers’ level of education: 1 = basic, 2 = secondary, 3 = university); and (3) IY-TCM training and delivery-related variables (IY-TCM training level: 0 = at university level and 1 = at local community level) and intervention professionals (0 = teachers, 1 = not teachers).

Non-significant moderation effects are not presented. Significant moderation effects were found for *children’s social and emotional skills* considering children’s behavior (for self-management/compliance and peer-relations/empathy), mothers level of education (for self-management/compliance) and level of children’s schooling (for peer-relations/empathy).

#### Children characteristics

**Figure 2:**
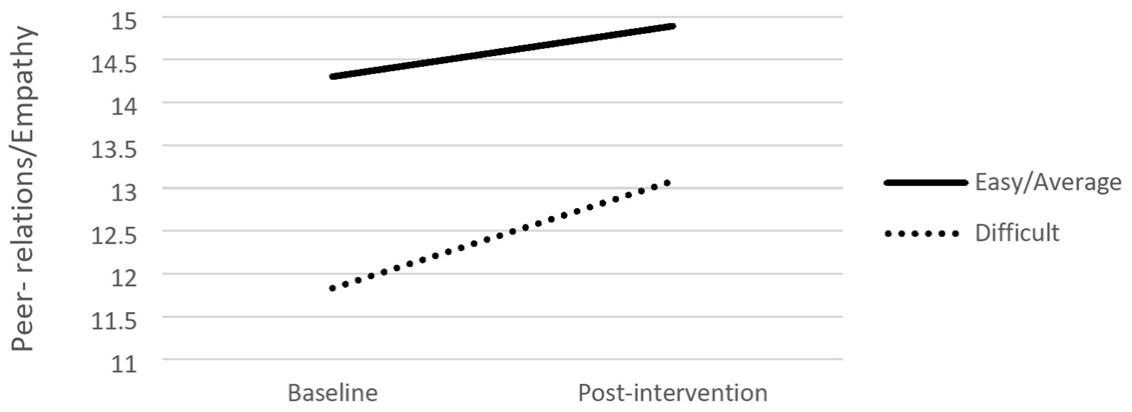
Moderation effect of children’s behaviour and peer-relations/empathy.

**Figure 3:**
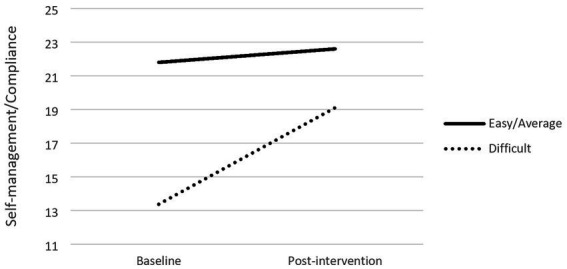
Moderation effects of children’s behaivour and self-management/compliance.

The evaluation of children as “easy/average” or “difficult” by their teachers was a significant moderator of the change of self-management/compliance and peer-relations/empathy skills. Indeed, regarding changes in peer-relations/empathy due to the intervention, results showed that children’s difficulty (*b* = −0.33) was significantly associated with changes in peer-relations/empathy scores (*R*^2^ = 03, *F*(1,506) = 18.02, *p* < 0.001). Conditional effects showed that effects were different between children assessed as “easy/average” (*b* = −0.59, *p* < 0.001) and those identified as “difficult” (*b* = −1.25, *p* < 0.001), with the latter group showing higher changes (cf. Figure 2). A similar effect was found regarding self-management/compliance. Results showed that the evaluation of children as “easy/average” or “difficult” by their teachers (*b* = −0.47) was significantly associated with changes in self-management/compliance scores (*R*^2^ = 0.19, *F*(1,503) = 19.39, *p* < 0.001). Conditional effects showed that effects were different between children evaluated as “easy/average” (*b* = −0.79, *p* < 0.001) and as “difficult” (*b* = −1.73, *p* < 0.001), with, again, the latter group showing higher changes (cf. Figure 3).

#### Mother’s level of education

**Figure 4:**
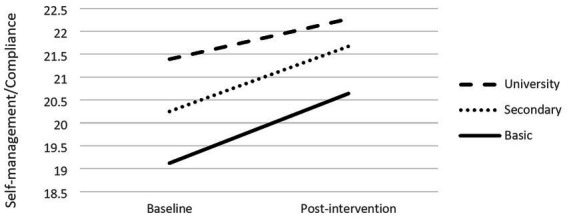
Moderation effects of mother’s level of education and self-management/compliance.

Mother’s level of education (*b* = 0.34) was also a significant moderator of changes in children’s self-management/compliance behavior (*R*^2^ = 12, *F*(1,391) = 5.45, *p* = 0.02). Conditional effects showed different slopes between mothers with basic (*b* = −1.49, *p* < 0.001), secondary (*b* = −1.21, *p* < 0.001) and higher (*b* = −0.93, *p* < 0.001) education, with the first two groups showing higher changes (cf. Figure 4).

#### Children’s level of schooling

**Figure 5:**
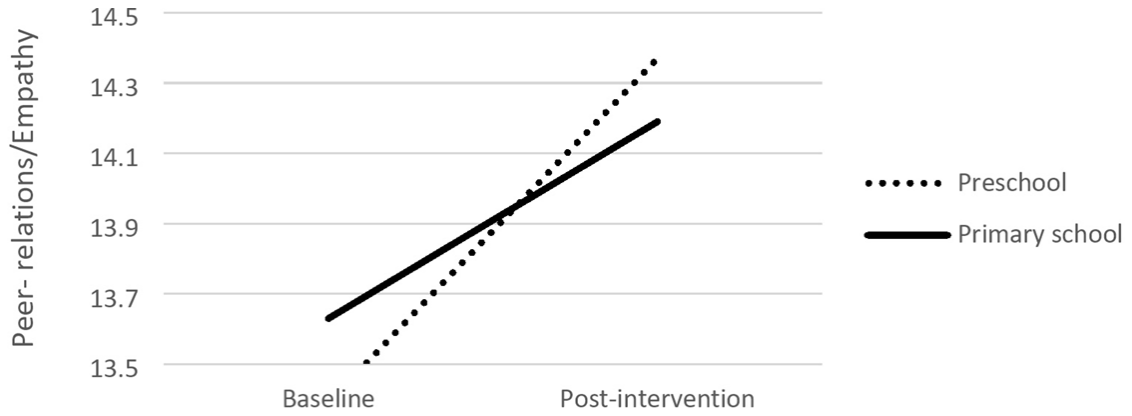
Moderation effects of children’s school level and peer-relation/empathy.

Finally, children’s level of schooling (*b* = 0.41) was also a significant moderator of changes in children’s peer-relations/empathy behavior (*R*^2^ = 12, *F*(1,506) = 5.45, *p* = 0.01). Conditional effects showed different slopes between preschool children (*b* = −0.98, *p* < 0.001) and primary school children (*b* = −0.56, *p* < 0.001), with the former showing higher change in peer-relations/empathy behavior (cf. Figure 5).

## Discussion

The Academias Gulbenkian do Conhecimento initiative provided a unique opportunity to understand the impact of the IY-TCM program on children’s social and emotional competence and skills when delivered on a large scale as an universal classroom-based intervention in the real world, and to understand how some variables (related with the children, the school-based professionals who deliver the program, and the type of group-leaders training) moderate that impact.

All the impact results found represent improvements in the desired directions, however with small effect sizes, and they confirm results of previous efficacy studies (RCT or quasi-experimental designs) where the IY-TCM was implemented as a stand-alone intervention, in other countries including in Portugal.

*The significant increase in social skills* as reported by teachers, in both dimensions assessed (one more related with self-regulation and compliance and the other with peer-relationships and empathy), is in line with the results found in other studies, as in the one conducted by Baker-Henningham et al. (2018) in a low-income country, Jamaica, with a sample of preschool children considered by their teachers as having the highest level of conduct problems in the classroom. However, unlike our study, the effect sizes found were high, perhaps because it was a high-risk sample with more space for improvement. Also relevant is the case from Norway, Fossum et al. (2017), which examined a universal sample of kindergartens from 3 to 6 years-old children, including a sub-sample of children who scored at or above the 90th percentile on aggressive behavior at baseline, and also found significant improvements in social skills based on teachers’ reports. However, small effect sizes were reported for the universal sample, as in our study, and higher for the behavior risk sub-sample. In Portugal, Vale (2012), in a universal sample of preschool children, and Seabra-Santos et al. (2018) with a sample of preschoolers from low-income areas, found the same type of improvement. The effect sizes reported in the Seabra-Santos et al. study (Seabra-Santos et al., 2018) are also small, yet they indicated that the children who benefited more from the intervention, in terms of social skills, are those with lower social skills at baseline and coming from families in economic need. In the recent meta-analysis conducted by Korest and Carlson (2022), where most of the previous studies we have just described were also included, as well as others conducted in other countries (United Kingdom, Ireland, New-Zealand and the United States), the efficacy of the IY-TCM is confirmed as a stand-alone program concerning the increase of prosocial behavior for teacher-rated reports, although with small effects sizes.

*Problem behaviors* were also assessed in our study using teacher-reports, and as for social skills, significant improvements were found with a reduction after the intervention.

In Baker-Henningham et al. (2018), significant reductions in teacher-reported behavior difficulties were also found and with medium effect sizes. The same reduction was observed in the Seabra-Santos et al. (2018) study, however without the differential impact found for social skills as described above. Fossum et al. (2017) also reveals a positive impact in the teacher-reported behavior difficulties in the universal sample, but for the high-risk group of children none of the reduction was significant at the 0.05 level. In a mixed methods systematic review, Nye et al. (2019) reported a small, statistically significant effect (using observation and teachers-report measures) of the IY-TCM on reducing child conduct problems, but only for high-risk conduct children. In the recent meta-analysis from Korest and Carlson (2022), small positive effects were found on children’s externalizing behaviors, with larger effect sizes for higher risk children (i.e., children with behavior problems above the clinical range as defined by the study).

One of the goals of the FCG academies is to improve *problem solving* defined as the way the child “realistically assesses problems, looks for alternatives, decides and implements solutions using creativity and logical thinking, keeping in mind the consequences on self and others” (see footnote 2). In our study the impact of the IY-TCM on children’s social problem-solving skills was assessed with a task administered via a one-on-one interview format. Our results provide consistent evidence of the positive impact of the IY-TCM program on the three indicators assessed, as statistically significant effects were found for the three changes analyzed. The three effect sizes were small, ranging from 0.19 to 0.25, however the highest effect size was obtained for the proportion of positive solutions compared with the other two. The assessment of the IY programs’ impact in social problem-solving skills is more usual when the IY programs for children are implemented versus when the programs used are directed at parents and teachers, which is one of the reasons why this outcome is not reported in the IY-TCM meta-analysis conducted by Korest and Carlson (2022). For instance, Williams et al. (2019) developed a RCT in primary schools where the universal IY Classroom Dinosaur School program was delivered by teachers to at risk children and where teachers were already trained in the IY-TCM. According to the results, improvements in the problem-solving knowledge of children, as evaluated by the Wally Problem Solving measure, were found in the intervention condition, compared to children in the control condition, with medium effect sizes for prosocial (ES = 0.39) and for agonistic (ES = 0.41) solutions.

Therefore, our results provide broad support as to the effectiveness of the IY-TCM, when implemented as a universal school-based program, on a large-scale and in the real world, as they yield significant improvements across the different variables assessed, that is, those related with children’s social and emotional competence, including social problem-skills. These results are in line with the seven socio-emotional competencies the Academias Gulbenkian do Conhecimento initiative sought to improve in children. However, to effectively reduce the gap between research and practice in education it is not enough to simply assess the impact of the intervention in the real world. According to Shonkoff (2017), we need to know not only whether the interventions achieve the intended effects, but also in what contexts, for whom and how. In order to answer the last two questions, moderation effects were examined for all the outcome variables.

Non-significant moderation effects were found when considering the level of IY-TCM training and the professional background of the professionals who delivered the intervention as moderators, meaning that the intervention was effective regardless the conditions. Concerning the IY-TCM training, the sessions at the university level involved experienced group-leaders from whom we could expect more adherence to the intervention’s components. Also, both are trained as psychologists and their clinical training could contribute to the development of skills central to the collaborative process and in the development of therapeutic alliance, which research about the role of the group-leaders of the IY Basic program for parents in Portugal highlights as central in the process of change (Leitão et al., 2022). Likewise, in Ireland the IY-TCM training to primary school teachers is offered by educational psychologists from the National Educational Psychology Service, as part of their continuing professional development (Davey and Egan, 2021). However, in a qualitative study about the teachers’ views on the acceptability and implementation of the IY-TCM in UK primary schools, the professional qualification of group-leaders (e.g., psychologist) was not indicated as important (Allen et al., 2022). Rather, they value group-leaders who are welcoming, supportive, open, friendly, non-judgmental or patronizing, who recognize them as experienced teachers, encourage them actively to value and support each other (Allen et al., 2022). The model of training and close supervision offered by the university team to local community group-leaders thus gave them the opportunity to develop those competences central in the collaborative process. The in-built fidelity tools of the IY-TCM program and all the materials (manuals, DVDs, books, and other items) provided to local group-leaders also served to increase the fidelity of implementation (Hutchings and Williams, 2017). Additionally, local group leaders had the opportunity to establish partnerships with local schools, school leaders and teachers and adequate the implementation to the needs of the participants in a more significant way. Furthermore, because they work at the local level, they can support teachers in a more personalized and intensive way and not be dependent on external support. Also, the teachers in the group can work with local peers and construct a stronger and sustainable community of support, considered by teachers themselves as one of the most important aspects of IY-TCM (Allen et al., 2022). Therefore, both training conditions had strengths that could explain why both are equally effective in our study.

Considering the professionals who implemented the intervention in classrooms, the non-significant moderation effects found indicate that the intervention was equally effective when delivered by teachers or by other professionals who work with children in the classroom. Durlak et al. (2022), in their review of 12 meta-analyses of universal, school-based social and emotional learning (SEL) programs, from pre-school to high-school, reported mixed results related with the type of professional who delivered the intervention, when they compared teachers with researchers. In our study all non-teacher professionals were like their teaching counterparts in that they also held a university degree and were experienced in working with children in a regular basis in their classrooms via planned activities with a focus in the socio-emotional development. They all attended the training at the university level by two experienced and qualified group-leaders. Our findings support the author’s assumption that the IY-TCM program can be implemented not only by teachers but also by other professionals working in educational environments (Webster-Stratton, 2011a).

When we move our focus to the variables of the children and the mother (initial behavior as reported by their teachers, children’s level of schooling, and mother’s level of education) the moderation results are mixed with respect to the outcome variable analyzed. According to our results, no significant moderation effects were found for teacher-reported behavior difficulties (measured with the SDQ), nor for social problem-solving strategies used by children (measured by the Wally test). In fact, when considering these outcome variables, we observed that all the children benefit similarly from the IY-TCM program.

However, significant moderation effects were found for the social skills as reported by teachers considering children’s initial behavior, children’s level of schooling and mother’s level of education. When initial behavior was taken as the moderator, significant effects were found both for self-regulation/compliance and for peer-relations/empathy, with children assessed as difficult showing more benefits from the intervention when compared to the ones assessed as easy/average. These results replicate the ones of previous research with Portuguese disadvantaged preschoolers (Seabra-Santos et al., 2018), which pointed out that the initial behavior risk was a moderator of the IY-TCM impact, with children at higher risk at baseline benefitting more from the intervention. As in the present study, the moderation effect found was only significant for social skills but not for behavior problems. Both results are in line with the Korest and Carlson (2022) meta-analysis: initial severity of child behavior is a moderator of program effects; and the effect sizes are higher for prosocial outcomes compared to externalizing behavior problems. One explanation for the higher impact on the prosocial behavior result could be the strong emphasis the program places on positive behavior. Thus, the theoretical foundation of the IY-TCM, expressed in a “teaching pyramid,” is that the teacher focuses first on increasing positive behavior rather than on reducing negative behavior (Webster-Stratton, 2012). As for the moderation effect of the severity of the initial child behavior, a possible explanation may have to do with a central tool of the program: the “individualized behavior plans” (Webster-Stratton, 2011b). Those plans are developed and applied by teachers with those children who pose the most behavioral challenges in classroom and the same intervention logic mentioned before is followed: start by increasing positive behaviors and only then, and if necessary, resort to strategies to reduce negative behaviors. As so, the development of a behavior plan for a difficult child in their classrooms is part of the teacher’s tasks during the training delivered in our study, and in supervision those plans are discussed and developed to respond to the child’s needs in a more effective way. Also, in our study teachers chose one of the two children they had indicated as difficult (two of the six children who were evaluated in the class) to be the target of their plan and this could be another reason that contributed to the results we found: the children who benefit more are the ones the teachers initially selected as difficult.

Other significant moderation effect found indicates that children from preschools took more from the intervention when compared to primary school children in terms of peer-relationships and empathy. We may be facing an age effect, and if so, our results are in line with the results found in five meta-analysis of SEL interventions reviewed by Durlak et al. (2022): younger children benefited more than older ones. However in the other six meta-analyses the authors reviewed, age was not found to be a significant moderator. Qualitative studies with the IY-TCM reported that some teachers felt the program was more suitable for younger children (4–6 years old as compared to 7–11 years old), and that some contents (e.g., the use of social coaching and descriptive comments) did not work well with older children (Allen et al., 2022). Concerning the Portuguese context, we may also hypothesize that primary school teachers, when compared to their preschool counterparts, lack the time, and at times the motivation, to implement the IY-TCM strategies, more directly focused on social and emotional development, in their classrooms, because their focus is more on cognitive learning. Therefore, conflict with the curricular goals is stronger in the primary school context compared to preschool context, where teachers have more autonomy to manage and choose the activities to develop in their classrooms, as they only have to follow curricular guidelines, and the emphasis on socio-emotional skills is stronger than in primary schools.

Finally, a significant moderation effect identified is directly related with self-regulation and compliance: children with mothers with basic or secondary education experience greater changes in self-regulation and compliance (but not in peer-relations/empathy) compared with children whose mothers have a university degree. This result is also in line with Seabra-Santos et al. study (Seabra-Santos et al., 2018), who reported that children who gained more from the intervention, with respect to social skills, were those coming from families in economic need. Low income and low level of education are both markers of low socioeconomic status (SES) (Berry et al., 2022).

### Strengths and limitations

Our results provide promising evidence that the IY-TCM—implemented as an universal school-based program in the real world, delivered by teachers or other school-based professionals, trained by existing staff in community services or by researchers from a university, with close supervision and support by a qualified and experienced team in the IY programs – yields significant improvements in different variables related with children’s socio-emotional and behavioral competence, benefiting those who exhibit more need: children with more difficult behavior and children whose mothers are less educated. These differential results thus contradict the Matthew effect, a hypothesis proposed to explain differential effects of interventions, which suggests that children who start with less disadvantage and higher skills are those who will benefit more because they are better equipped to take advantage of the learning opportunities and have more capacity to build on their initial skills. On the contrary, our results reinforce the compensatory hypothesis based on the higher risk and greater room for improvement that some children demonstrate (McClelland et al., 2017).

However, we must keep in mind that certain limitations exist in our study. An initial and broader limitation has to do with the absence of a systematic assessment and/or analysis of the implementation effectiveness. Considering the “Implementation Outcomes Taxonomy” (Proctor et al., 2011), acceptability, adoption, appropriateness and feasibility were assessed at program participants’ level and considering teachers and group-leaders’ perceptions expressed in the IY-questionnaires. Focus groups were conducted with teachers and group-leaders at the end of the first year of the academies. However, that data haven’t been analyzed so far. Future studies also need to assess and control systemic variables that could impact not just the success of the intervention but also the success of the implementation (Allen et al., 2022) at diverse levels, such as the individual (e.g., personal and professional competencies of group-leaders and teachers), the contextual (e.g., internal and external support, learning climate, staff, leadership) and the social (e.g., popularity of school-based SEL programs, educational policy) (Hagermoser Sanetti and Collier-Meek, 2019; Durlak et al., 2022).

Another limitation is the absence of a control group specifically for the implementation in primary schools, where the IY-TCM effectiveness has not yet been demonstrated in the Portuguese context. An RCT with primary school teachers, accompanied by a qualitative study, could help to understand why primary school teachers benefited less from program participation, compared to preschool educators, as shown in our study.

As for the measure used to assess social and emotional skills in order to achieve a common measure for both preschoolers and school aged children, 10 items were retained from two different questionnaires, one to be answered by primary school teachers and other by their preschool counterparts. The author is the same for both measures and good internal consistency levels were obtained for both set of items: Self-Management/Compliance, and Peer Relations/Empathy, at baseline and at post-intervention. However, more psychometric studies need to be developed with this new adaption, which has the strength of being usable to evaluate children at both levels of schooling.

Regarding the measures used, it is important to note that the Wally Problem Solving test was applied here in Portugal for the first time; it was included in the protocol for evaluating the implementation of IY-TCM as well for the first time. However, because of the absence of previous studies in Portugal, more studies are needed. Also, the degree of difficulty in the child’s behavior at baseline was established based on their teachers’ reports and not on a standardized measure, which can also be seen as a limitation of this study.

Finally, considering that the intervention was implemented in several schools, there may be some variability across schools that was not accounted for. Indeed, it might well be noted that certain results are attributable to the characteristics of the school itself, thus representing a source of bias and one which our statistical analysis did not take into account.

## Conclusion

The implementation model described in this paper meets the needs of the FCG via the Academias Gulbenkian do Conhecimento project. We demonstrated how a team of researchers linked to a university and with extensive experience in research and dissemination of EBP was able to develop and implement a model that not only contributed to reducing the gap between research and practice, but also proved to be able to promote changes in social and emotional competencies related to the mission of the academies. The existence of a “university champion” that shows leadership and had access to the decision makers (the funder) is considered by some authors as a critical element contributing to successful implementation (Hutchings and Williams, 2017). The “local champions” who led level 1 academies, and which worked closely with the coordination team from the university, enhanced the conditions for successful implementation and reinforced the guarantee of sustainability. The proportionate fidelity of the implementation, ensuring that all academies used the same high dosage (42 h) but with different application formats (monthly, fortnightly) and modalities (face-to-face, online or mixed) may have been one of the factors that contributed to its acceptability, adoption and appropriateness (Proctor et al., 2011). At the same time, this also shows how it is possible to make small adaptations to programs transported from other countries without distorting them yet still maintaining their effectiveness (Nye et al., 2019).

Findings from our study support expanding the IY-TCM model of implementation and training adopted, along with research that could respond to the limitations of our study. Pilot cost-effectiveness studies also need to be done in order to test the feasibility of including this model in Portugal’s national system of continuing professional development for teachers. This is an important step on the path to achieving desirable educational and social equity and to maintaining the schools’ and the teachers’ central position in the promotion of not only the emotional and social development of children but also their mental health and well-being, qualities which are essential in society’s efforts to achieve some of the 17 United Nations Sustainable Development Goals (SDGs) (e.g., SDG 1—No Poverty; SDG 3—Good Health and Well-Being; SDG 4—Quality Education; SDG 10—Reduce Inequalities).

## Data availability statement

The raw data supporting the conclusions of this article will be made available by the authors, without undue reservation.

## Ethics statement

Ethical review and approval was not required for the study on human participants in accordance with the local legislation and institutional requirements. Written informed consent to participate in this study was provided by the participants’ legal guardian/next of kin.

## Author contributions

MG: conceptualization, implementation coordinator, group-leaders training, teachers training, supervision, methodology, writing—original draft, and writing—review. MS-S: conceptualization, assessment coordinator, methodology, writing—original draft, and writing—review. JR: assessment, data collection, and writing—review. MP: teachers training and supervision, assessment, data collection, and writing—review. TH: teachers training and supervision and writing—review. AA: group-leaders training and supervision and writing—review. MM-R: statistical analysis, writing—original draft, and writing—review. All authors contributed to the article and approved the submitted version.

## Footnotes

^1^ https://gulbenkian.pt/en/the-foundation/the-foundation/

^2^ https://gulbenkian.pt/academias/competencias-alvo/

^3^ https://gulbenkian.pt/academias/publications/
